# Optimization and Characterization of Aspirin Encapsulated Nano-liposomes

**Published:** 2018

**Authors:** Samira Khodayar, Hassan Bardania, Seyed Abbas Shojaosadati, Fatemeh Bagheri

**Affiliations:** a *Biotechnology Group, Department of chemical engineering, Tarbiat modares University, Tehran, Iran. *; b *Cellular and Molecular Research Center, Yasuj University of Medical Sciences, Yasuj, Iran.*

**Keywords:** Cholesterol, Sodium Lauryl Sulfate (SLS), Aspirin encapsulated nano-liposomes (AS-NL), cytotoxicity

## Abstract

Resistance to aspirin and its cytotoxicity significantly limits its therapeutic applications. Nano-liposomal encapsulation of aspirin can reduce its cytotoxicity. In this study, aspirin encapsulating nano-liposomes (AS-NL) was prepared and its performance in drug delivery and also cytotoxicity was evaluated. The effects of two independent variables including number of freeze/thawing cycles and concentration of aspirin on encapsulation efficiency was investigated using response surface methodology (RSM). A drug profile release was obtained by AS-NL. The concentration of cholesterol as effective for liposome stability and sodium lauryl sulfate (SLS) as a drug release facilitator was also optimized using RSM. The maximum aspirin encapsulation efficiency (41.44%) and drug release (33.92%) was obtained for 0.514 mg cholesterol and 0.007 mg SLS used for liposome formulation. The morphology and size of AS-NLs were characterized using transmission electron microscopy (TEM) and dynamic light scattering (DLS). The stability of AS-NL was evaluated by measuring the size change of nano-liposomes during 21 days using DLS analysis. The stability of AS-NL during this period was acceptable. The cytotoxicity test of AS-NL by MTT test reveals the cytotoxicity of aspirin can be reduced by using liposome encapsulation.

## Introduction

Aspirin is the most widely used drug in the world (1) and approximately 36% of the United States adults take aspirin for cardiovascular disease prevention (2). The therapeutic usage of aspirin such as acute inﬂammation and pain, fever, cardiovascular diseases, heart attacks and stroke, pregnancy complications, cancer especially colorectal, diabetes, angina, thrombosis, thromboprophylaxis in orthopedic surgery, pericarditis, and Alzheimer’s disease, reﬂects its colorful achievements (3-6). Aspirin induces a long lasting functional defect in platelets, clinically detectable as a prolongation of the bleeding time (7). In spite of extend use of aspirin in treatment of various diseases, it causes some limitations including some toxicities and gastrointestinal bleeding because of high dose aspirin therapy (8), resistance, and nonresponse to aspirin as the incomplete inhibition of platelet function of the patients treated with aspirin (4), and also reduced absorption of active aspirin due to inadequately low dose of aspirin and intake of proton pump inhibitors (3, 9-11).

Due to these challenges, aspirin microencapsulation and delivery using nano-carries are considered. Liposomes are one of the most suitable carriers for drug delivery applications due to their unique characteristics including: biocompatibility, biodegradability, self-assembly, low cytotoxicity, lack of immune system activation, ease of synthesis, scalability and capability to incorporate both hydrophilic and hydrophobic drugs (9-11). Liposomes as a drug delivery system can reduce cytotoxicity of chemical drugs and improve the therapeutic activity and safety of drugs mainly by delivering them to their site of action and by maintaining therapeutic drug levels for prolonged periods (12, 13).

Formulation parameters including lipid composition, vesicle size, lipid membrane ﬂuidity, surface charge, cholesterol content, and steric stabilization should be optimized to extend the therapeutic effect of liposomal drugs (10). Cholesterol plays a key role on fluidity, stability, and permeability of lipid membranes. Remarkably, the use of cholesterol derivatives, that is, glycosylated cholesteryl, enhances the selectivity of liposomes toward non-healthy cells (14). Also after intercalation with phospholipid molecules, cholesterol alters the freedom of motion of carbon molecules in acyl chain and minimizes phospholipid exchange and mobility and increases the stability of the bilayer to prevent drug leakage (10). Cholesterol incorporation increases the separation between choline head groups, eliminates Vander Waals interactions between hydrogen chains of fatty acids, and prevents liposome crystallization (15). 

The present study aimed to optimize the preparation of AS-NL and also evaluate the effect of cholesterol and SLS on aspirin encapsulation efficiency (AEE) in liposomes and its drug release profile. Formulated AS-NLs were characterized using transmission electron microscopy (TEM) and dynamic light scattering (DLS) analysis. The stability of nano-liposomes was assessed by evaluation of size change of nano-liposomes with employing DLS analyses after 3 weeks. Cytotoxicity of nano-liposomes was investigated utilizing MTT test on human umbilical vein endothelial cells (HUVEC) as a normal cell line. 

## Experimental

*Materials*

Aspirin was prepared from Toli Daru (Iran), and cholesterol (Chol) from Sigma Aldrich (USA). Distrearoylphosphatidylcholine (DSPC) was purchased from Avanti polar (USA). Human umbilical vein endothelial cell line culture (HUVEC) was purchased from Iran Pasteur Institute. DMEM was purchased from Gibco and Sodium Lauryl sulfate (SLS) and other chemicals were obtained from Merck (Germany). 

*Preparation of nanoliposomes*

Nanoliposomes were prepared using thin film method (21). Briefly, predetermined amounts (Tables 3, and 6) of DSPC, cholesterol, and SLS were dissolved in chloroform/methanol solution and added in a round bottom flask. The organic solution was evaporated to form a thin film of lipids using a rotary evaporator (IKA RV 10) under vacuum. The thin film was hydrated using Tris buffer (pH 7.4) during 2 h in water bath at 65 °C and 150 rpm to form liposomes. Liposomes were sonicated for 5 minutes with a probe sonicator (Geprufte Sicherheit UP400S, Germany). All experiments were carried out with a basis of 10 mM of DSPC.

**Table 1:** Experimental variables at different levels used in CCD.

**Name**	**Code**	**minus 1 Level**	**Center**	**plus 1 Level**	**minus alpha**	**plus alpha**
The number of Freeze/Thawing cycles	A	2	5.5	9	1	11
aspirin Concentration (mM)	B	6.5	10	13.5	5.05	14.95

**Table 2:** Factors and levels used in RSM

**Factors**	**Code**	**minus alpha**	**minus 1 Level**	**Center**	**plus 1 Level**	**plus alpha**
Cholesterol concentration (mg)	A	0	0.18	0.620	1.06	1.24
SLS concentration (mg)	B	0	0.003	0.010	0.017	0.02

**Table 3:** CCD of experiments for AEE optimization utilizing DX-7 software

Run	The number of freeze/thawing	Aspirin concentration (mM)	AEE (%)
1	6	14.95	27.5
2	6	10	29.6818
3	1	10	14.7075
4	2	13.5	16.9873
5	6	5.05	24.9684
6	9	13.5	33.8852
7	2	6.5	16.9784
8	6	10	30.6818
9	6	10	28.9818
10	11	10	32.1561
11	6	10	27
12	9	6.5	33.3763
13	6	10	27

**Table 4:** ANOVA for response surface Quadratic Model

**Source**	**SS**	**DF**	***p*** **-value**
Model	469.97	5	<0.0001
A	420.09	1	<0.0001
B	2.1	1	0.4372
AB	0.063	1	0.891
A^2^	43.48	1	0.0072
B^2^	8.4	1	0.1433
Residual	21.65	7	
Lack of Fit	10.91	3	0.3763
Pure Error	10.75	4	
Cor Total	491.62	12	

SS = Sum of Squares; DF = Degree of Freedom

**Table 5:** Result of confirmation test for AEE

**Confirmation Experiment (%)**	**Prediction**	**95 % CI low**	**95 % CI high**
33.26 ± 1.3	33.47	31.51	35.44

**Table 6:** Central composite design matrix for independent variables and their response values

**Run**	**Cholesterol Concentration (mg/mL)**	**SLS Concentration (mg/mL)**	**AEE (%)**	**Drug Release %**
1	0.620	0.010	41.33	33.91
2	1.058	0.017	40.37	27.25
3	0.000	0.010	41.06	29.69
4	0.062	0.000	41.73	26.51
5	0.062	0.010	41.30	34.34
6	0.062	0.010	40.30	34.50
7	1.240	0.010	40.58	29.36
8	0.062	0.020	41.35	23.73
9	0.182	0.003	41.23	29.67
10	0.062	0.010	41.32	34.24
11	0.182	0.017	40.80	27.32
12	1.058	0.003	41.02	25.50
13	0.062	0.010	41.29	33.97

**Table 7:** ANOVA of AEE and drug release

**Source**	**DF**	**AEE (%)**		**Drug Release (%)**	
		**SS**	***p*** **-Value**	**SS**	***p*** **-Value**
Model	5	1.96	0.0002	171.75	< 0.0001
A-Chol	1	0.21	0.0072	2.76	0.0497
B-SLS	1	1.16	< 0.0001	2.57	0.0561
AB	1	0.013	0.3943	4.22	0.0220
A^2^	1	0.48	0.0008	37.01	< 0.0001
B^2^	1	0.16	0.0145	141.29	< 0.0001
Residual	7	0.11		3.44	
Lack of Fit	3	0.11	0.0001	3.19	0.0098
Pure Error	4	8.995E- 004		0.25	
Cor Total	12	2.07		175.19	
R^2^					0.95
Adjusted R^2^					0.91

DF = Degree of Freedom; SS = Sum of squares

**Table 8:** Confirmation test for predicted optimum condition

**Response**	**Prediction**	**95% CI low**	**95% CI high**	**confirmation experiment**
AEE%	41.4422	41.31	41.57	41.39
Drug Release%	33.9175	33.19	34.64	33.26

**Table 9:** Size changes of AS-NLs during three weeks storage period at 4 °C.

**Storage Time (week)**	**Size (nm)**	**PDI**
0	74.46	0.228
1	92.50	0.201
2	95.65	0.379
3	129	0.203

**Figure 1:**
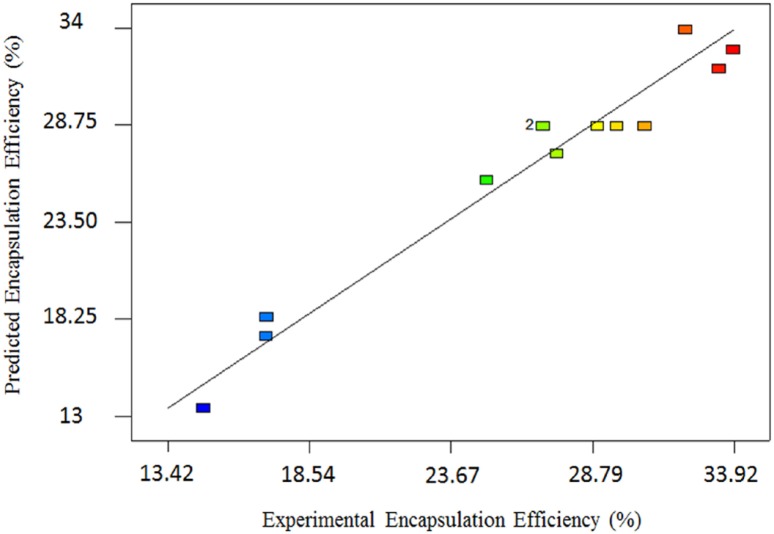
Actual vs. predicted values of AEE

**Figure 2:**
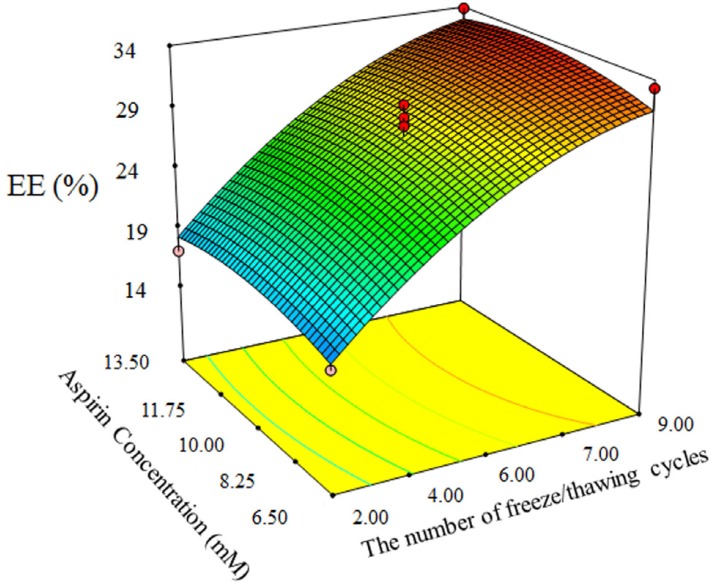
3D plot of interactive effect of aspirin concentration and number of freeze/thawing model on AEE.

**Figure 3:**
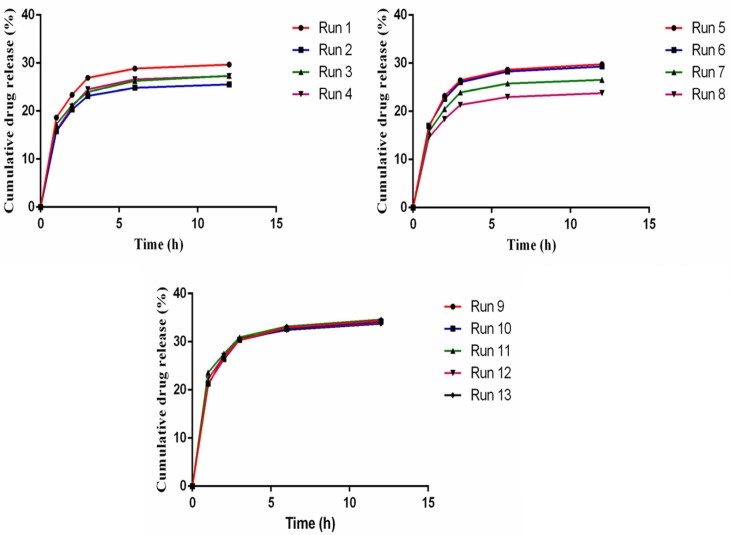
Aspirin release profile for designed experimentals (Table 6).

**Figure 4:**
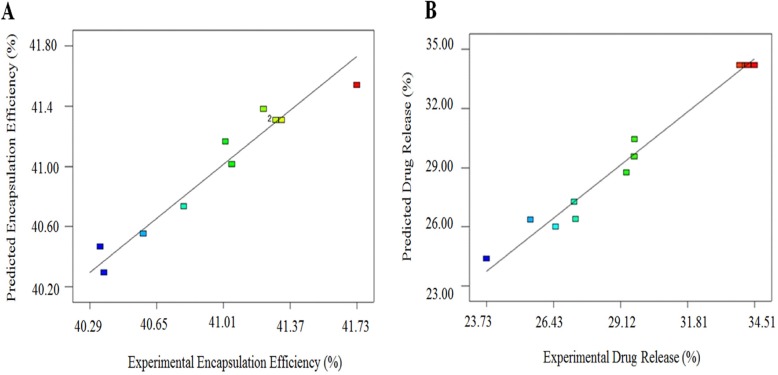
Predicted vs. actual for (A) AEE; (B) drug release

**Figure 5:**
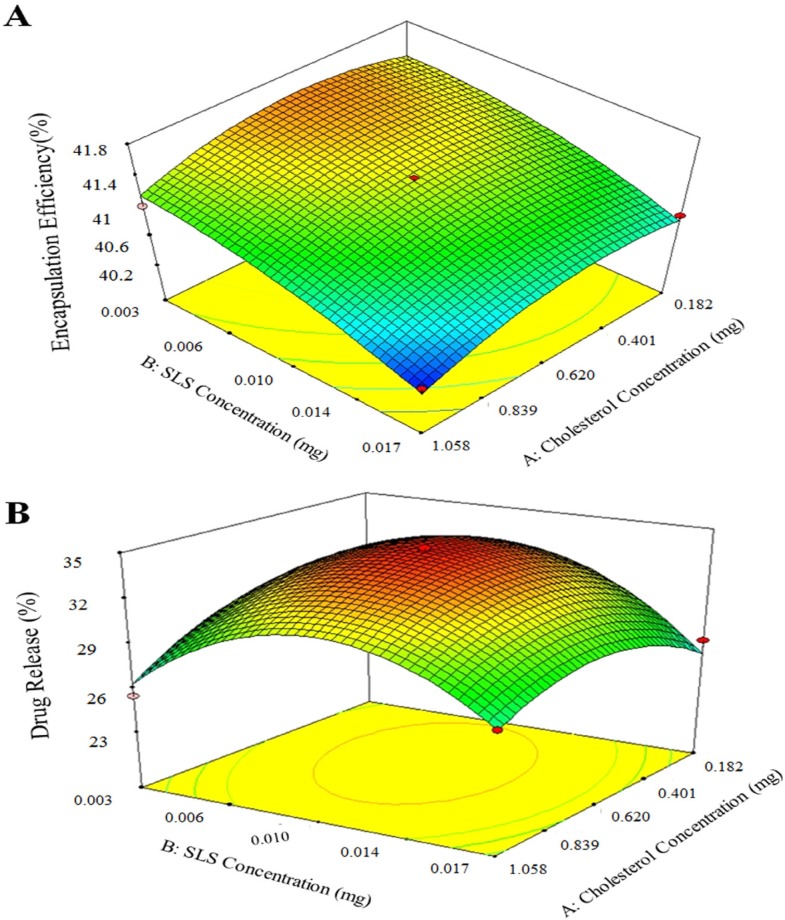
Response surface plots results of factors interactions on (a) encapsulation efficiency (%); (b) drug release (%).

**Figure 6:**
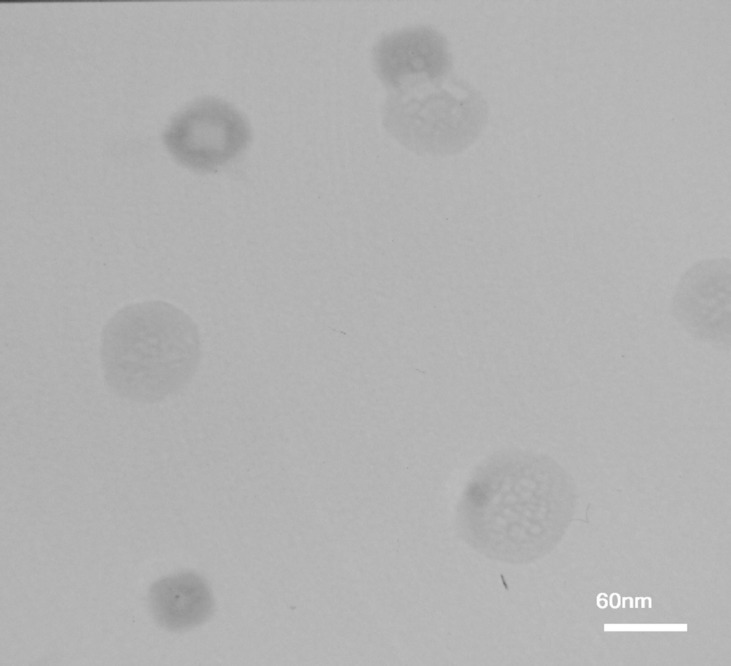
TEM images of AS-NL

**Figure 7:**
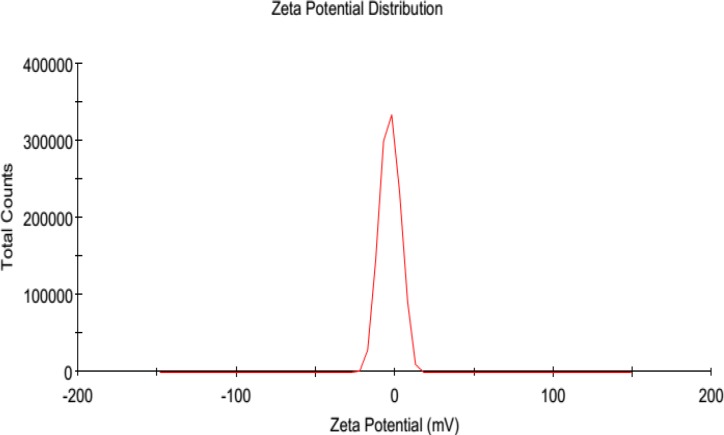
Size distribution of Aspirin encapsulated nano-liposomes

**Figure 8:**
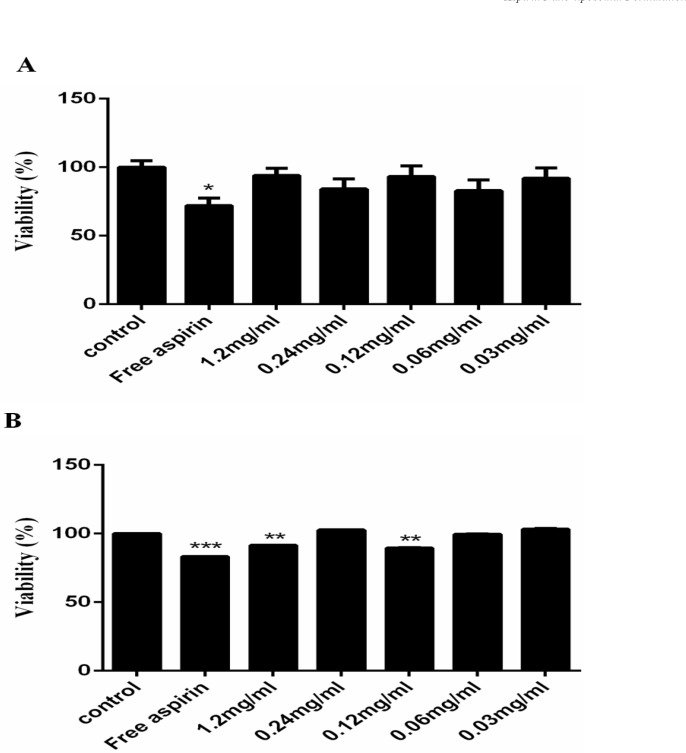
Cell viability after treatment with free aspirin (1.2 mg/mL) and different concentration of AS-NLs (A) after 24 and (B) 48 h

*AEE optimization*

Encapsulation of aspirin into nano-liposomes was performed using freeze/thawing technique. Various amount of aspirin and drug free nanoliposomes were mixed and the mixture was subjected to different freeze/thawing cycles (5 min at −196 °C and 5 min at 65 °C). Finally, entrapped drugs in nano-liposomes were separated from free ones using centrifugation (20000 rpm, 30 min). AS-NL was separated by centrifugation and the supernatants were collected to measure the non-encapsulated drug by HPLC. A reverse phase C18 column (Zorbax®, Hewlett-Packard, Sigma, Germany) was used as the stationary phase and separation was obtained using an isocratic mixture of acetonitrile:water with 20:80 (v/v) ratio containing 0.1 % phosphoric acid, with a constant flow rate of 1 mL/min and detection at 220 nm.

Optimization was carried out based on literature review, two important and independent factors of number of freeze/thawing cycles and aspirin concentration was used in the design of the experiments by RSM. The ranges and level of the variables are listed in Table 1.

The effect of independent variables was investigated through a thirteen designed experiments using a modified quadratic model (equation 1). 

Y = β_0_ + β_1_X_1_ + β_2_X_2_ + β_11_X_1_^2^ + β_22_X_2_^2^ + β_12_X_1_X_2_                    (1)

where Y is the response variable, X_1_ and X_2_ are the independent variables, β_0_ is the intercept coefficient, β_1_ and β_2_ are the linear coefficients, β_11_ and β_12_ are quadratic coefficients and β_12_ represent interaction coefficient. Finally, the validation of the model was assessed by performing the experiment at optimal factor levels and the experimental value of AEE was compared with the result predicted by the model. 

*Optimization of the effect of cholesterol and SLS on AEE and drug release using RSM*

A response surface method experimental design was constructed to derive experimental formulations for optimization of cholesterol (0-40% mole DSPC) and SLS (0-1% mole DSPC) concentrations to get the maximum AEE and drug release. The levels of the factors are shown in Table 2.

A central composite design (CCD) was used for modeling and the total number of trials was calculated as 2^k^ + n_a_ + n_0_, where n_a_ represents the axial points; n_0_ is the center points; and k is the number of independent variables. A factorial design by 2 factors and 5 central points was selected using Design-Expert (7.1.4) software. The behavior of the system was explained using the quadratic polynomial empirical model (equation 2) (16).

Y= α_0_ + α_1_X_1_ + α_2_X_2_ + α_11_X_1_^2^ + α_22_X_2_^2^ + α_12_X_1_X_2_                     (2)

where Y represents the response variable; α_0_ is the model constant; X_1_ and X_2_ are independent variables; α_1_ and α_2_ are linear coefficients; α_12_ is cross-product coefficients and α_11_ and α_22_ are quadratic coefficients. To ensure the validity of the model, experiment at optimal factor levels was carried out with triplicate and the experimental values for AEE and release were compared with the result predicted by the model. 

*Characterization of AS-NL and stability evaluation*

The size and morphology of AS-NLs were investigated using Transmission electron microscopy (TEM) and dynamic light scattering (DLS). Physical stability of nano-liposomes was evaluated by monitoring size change of nanoliposomes at 4 °C during 3 weeks (every week sampling). 

The polydispersity (PDI) of AS-NLs was obtained by diluting (1:50) of sample with SBF (pH 7.4). 

*In-vitro cytotoxicity assay of the AS-NLs*

The cytotoxicity of AS-NLs was assessed using MTT test on Human umbilical vein endothelial cells (HUVEC). Approximately 4 × 10^4^ cells were cultured in each 96-well plates containing DMEM and FBS (10%) and incubated at 37 °C for 24 h to allow cell attachment. The cells were treated with different concentration of AS-NLs (1.2, 0.24, 0.12, 0.06, 0.03 mg/mL) and MTT assay was performed after 24 and 48 h. The cells were cultured in absence of AS-NLs as control. After cell incubation with nano-liposomes, the medium was replaced with a 5:1 ratio of medium and MTT solution (5 mg/ml in PBS). The cells were incubated at 37 °C for 4 h until purple formazan crystals were formed. Then, 150 µL of DMSO was added to wells and the absorption at 570 nm was measured by ELIZA reader. The viability of treated and control cells was calculated using equation 3.

Cell viability %=(Optical density OD of the treated cellsOD of control cells)×100           (3)

## Results and Discussion

*AEE optimization*

A CCD and RSM were employed to optimize the number of freeze/thawing and aspirin concentration variables. A 2^2^ factorial design and (2×2) axial points with five replications at the center points lead to a total number of 13 experiments using Design-Expert 7.1.4 software (17).

Each factor was considered at five different levels and optimal ranges for the variables were coded to lie at ± 1 for factorial points, 0 for center points, and ± a for axial points. The CCD was employed to fit the model and shows complete design matrix of experiments and their responses (Table 3). Equation 4 is the final empirical model in coded terms obtained for AEE

EE%=+28.67+7.25A+0.51 B-2.5A2-1.5B2           (4) 

Where A and B are the number of Freeze/Thawing cycles and aspirin concentration respectively. The analysis of variance (ANOVA) for modified quadratic model for AEE is shown in table 4. ANOVA results show that the model is significant (*p < 0*.0001) and lack of fit is not significant. The high R^2^ and adjusted R^2^ are 0.96 and 0.93 respectively; these values approve indicating that the quadratic equation for the AEE is capable of representing the system under the given experimental conditions, and predicted and actual AEE were in agreement. Figure 1 shows a good agreement between the experimental and predicted data for AEE.

In addition, ANOVA shows that number of freeze/thawing cycles has significant influence on AEE (*p << 0.0001*), while aspirin concentration has not significant influence (Figure 2). The significant effect of number of freeze/thawing cycles is because of ice crystals formation during freezing of nano-liposomes. So that ice crystals forms pores inside of nano-liposomes shell and allow more drug entrap into nano-liposomes (18, 19), and subsequently heating above Tc cause restoring of phospholipid structure and reformation of liposome.

The optimum condition of number of freeze/thawing and aspirin concentration for maximum AEE was 9 cycles of freeze/thawing and 10.82 mM of aspirin concentration. 

The predicted optimum condition was validated by performing additional experiments in triplicate. In table 5 the result of experiment was conducted at optimum condition compared to predicted values by model. The result of analysis indicated that the experimental value was 32.26 ± 1.3 that is between CI low and CI high predicted values.

*The effect of cholesterol and SLS concentration on AEE and drug release*

Preliminary tests for aspirin release from AS-NL show low release (10 -15%), therefore SLS as a surfactant was used to increase the fluidity of phospholipid layer and the release. An RSM experimental design was applied to find the most suitable amount of cholesterol and SLS to get the maximum AEE and drug release. The experiments designed for two factors and their results are presented in table 6. Figure 3 shows the result of drug release response of designed experiments.

The data was analyzed to identify the signiﬁcance of the factors, their optimal values and interactions using analysis of the variance (ANOVA) (Table 7). The Results revealed that the model is significant and ″*p*-value″ of factors, A^2^ and B^2^ were lower than 0.05, which means that they had significant effect on AEE (%) and drug release (%). The adjusted R^2^ for the predictive model was 0.91 for encapsulation efficiency and 0.97 for drug release; this approve suggested that the experimental validated the selected equation. Adding a variable to the model always raises R^2^ regardless of whether the additional variable is statistically significant or not. Therefore, adj-R^2^ is used to validate the model. In addition, the values of R^2^ and adjusted R^2^ are similar, therefore non-significant terms have not been included in the model.

Figure 4 shows the plot of the residuals against the predicted responses for (A) encapsulation efficiency (%) and (B) drug release (%). The evenly distribution of the points for both models indicates that the model is sufficient. Equations 5 and 6 are the regression equations of the responses in terms of actual factors.

AEE% = +41.31 -0.16 A -0.38 B – 0.056 AB -0.26 A^2^ – 0.15 B^2^                     (5)

Drug release % = +34.19 -0.59 A -0.57 B + 1.03 AB – 2.31 A^2^ – 4.51 B^2^                     (6)

The results of 3D plots for cholesterol content increase as shown at Figure 5 show that the cholesterol content enhances the aspirin loading. Because entrapment increase due to aspirin molecule has hydrophobic and hydrophilic parts, therefore it interacts with cholesterol. Of course, increase of cholesterol over optimal value causes encapsulation efficiency decrease due to the excessive filling spaces between side chains of DSPC. On the other hand, SLS addition decreases drug loading as it raises fluidity of the liposome membrane. Similar trend was obtained for aspirin release from AS-NLs. As expected, SLS plays a key role in drug release increase, however more SLS increase resulted in drug release decrease.

The predicted optimum conditions suggested by the model were at 0.514 mg cholesterol and 0.007 mg SLS to obtain 41.44% AEE and 33.92% drug release. In order to validate the reliability of the model equations, conformation test was carried out under the optimum conditions in triplicate (Table 8). The results were in the predicted range of software. Therefore, the optimization of AS-NL by response surface methodology was practical and reliable.

*Characterization of nano-liposomes*

The results of TEM analyses for characterizing nano-liposomes showed that the size of nano-liposomes was approximately 80 nm (Figure 6).

The nano-liposomes were subjected to the storage stability study for a period of 3 weeks. The average size and distribution of particle was measured by DLS analysis. The results are shown in table 9 and Figure 7. The high stability and low aspirin release from nano-liposomes are related to the chain length of phospholipids. The phospholipids with long chain length have high transition temperature. High transition temperature (55 °C) of DSPC cause physical stability of nano-liposomes in lower temperatures (20).

*In-vitro cytotoxicity*

The cytotoxicity of free aspirin (1.2 mg/mL) and AS-NLs in presence of HUVEC cells was examined by MTT assay at ﬁve different drug concentrations (1.2, 0.24, 0.12, 0.06, 0.03 mg/mL) for 24 and 48 h. The results are showed in Figure 8 in which the free aspirin exhibit significant cytotoxicity on these cells, which AS-NL cytotoxicity was not considerable after 24 h. Nano-liposomes are biocompatible nano-carrier and have not cytotoxicity on normal cells and the cytotoxicity of chemical drugs can be reduced by liposome encapsulation (21). The previous research approve that aspirin has more cytotoxicity on cancerous cells than normal cells (22). 

The viability of cells treated with free aspirin was 70%, but AS-NL did not significantly decrease the cell viability (Figure 8). 

## Conclusion

In this study, we found the profound effect of cholesterol and SLS on properties of AS-NL specially loading and release profile. The optimum condition for AS-NL was obtained in which the delivery system improved considerably. The cytotoxicity evaluation of AS-NL reveals the potential application of AS-NL as a new delivery system without any side effects for patients having limitation in taking free aspirin. 
